# Predictive value of daily living score in acute respiratory failure of COPD patients requiring invasive mechanical ventilation pilot study

**DOI:** 10.1186/1471-2466-12-66

**Published:** 2012-10-18

**Authors:** Ketty Langlet, Thierry Van Der Linden, Claire Launois, Caroline Fourdin, Philippe Cabaret, Nadia Kerkeni, Coralie Barbe, François Lebargy, Gaetan Deslée

**Affiliations:** 1 Service d’Urgences et de Réanimation médicale polyvalente, Hôpital Saint Philibert, Lomme, France; 2 Unité d’Aide Méthodologique, Pôle Recherche et Innovations, Hôpital Robert Debré, CHU de Reims, France; 3 Service de Pneumologie, INSERM UMRS 903, Hôpital Maison Blanche, CHU de Reims, France

**Keywords:** Chronic obstructive pulmonary disease, Acute respiratory failure, Mechanical ventilation weaning, Mortality, Activities of Daily Living score

## Abstract

**Background:**

Mechanical ventilation (MV) is imperative in many forms of acute respiratory failure (ARF) in COPD patients. Previous studies have shown the difficulty to identify parameters predicting the outcome of COPD patients treated by invasive MV. Our hypothesis was that a non specialized score as the activities daily living (ADL) score may help to predict the outcome of these patients.

**Methods:**

We studied the outcome of 25 COPD patients admitted to the intensive care unit for ARF requiring invasive MV. The patients were divided into those weaning success (group A n = 17, 68%) or failure (group B n = 8, 32%). We investigated the correlation between the ADL score and the outcome and mortality.

**Results:**

The ADL score was higher in group A (5.1 ±1.1 vs 3.7 ± 0.7 in group B, p < 0.01). Weaning was achieved in 76.5% of the cases with an ADL score ≥ 4 and in 23.5% of the cases with an ADL score < 4 (p < 0.05). Pulmonary function test, arterial blood gases collected during period of clinical stability and at admission and nutritional status were similar in both groups. The mortality, at six months, was 36%. The ADL score was a significant predictor of 6-month mortality (80 with an ADL score <4, 20 with an ADL score ≥4, p < 0.01).

**Conclusion:**

Our pilot study demonstrates that the ADL score is predictive of weaning success and mortality at 6 months, suggesting that the assessment of daily activities should be an important component of ARF management in COPD patients.

## Background

The natural course of chronic obstructive pulmonary disease is characterized by recurrent episodes of respiratory failure sometimes requiring mechanical ventilation (MV). The use of non invasive ventilation (NIV) has dramatically reduced the need of endotracheal intubation (EI) [1], but EI remains required in case of failure of NIV or initially life-threatening acute respiratory failure (ARF). The prognosis of ARF in chronic obstructive pulmonary disease (COPD) patients requiring EI for MV is poor with a one-year mortality ranging from 30 to 50 [2], representing a difficult challenge for the clinicians. Several studies have shown the difficulty to identify clinical and functional parameters predicting the outcome of these COPD patients treated by invasive MV [3,4]. However, these studies have been conducted in 1980s-1990s, when NIV was not commonly used in acute exacerbation of chronic pulmonary disease (AE-COPD). Since NIV is nowadays largely used as first line treatment in severe AE-COPD and also in ARF whatever the cause, the severity of patients requiring invasive MV has increased [5]. In this study, we carefully analyzed the current characteristics of COPD patients requiring invasive MV for ARF and the correlations with weaning success or failure.

Our hypothesis was that a non specialized score as the activities daily living (ADL) score may help to predict the outcome of these patients. To test this hypothesis, we conducted a pilot study to investigate the correlation between the ADL score, clinical and pulmonary function parameters, and the outcome and mortality of COPD patients requiring invasive ventilation.

## Methods

### Study design

All COPD patients with ARF admitted to the intensive care unit (ICU) of the Saint Philibert Hospital in France were considered for inclusion. The criteria of inclusion was ARF requiring EI for MV in a patient with a diagnosis of COPD based on at least one pulmonary function test (PFT) performed during a phase of clinical stability before AE-COPD. The diagnosis of COPD was established on the basis of the Global Initiative for chronic Obstructive Lung disease (GOLD) consensus statement as a ratio of forced expiratory volume in one second over forced vital capacity (FEV_1_/FVC) of less than 0.7 after the administration of 200 μg of salbutamol [6].

Exclusion criteria were neuromuscular disease, restrictive pulmonary disorder defined by PFT showing a predicted total lung capacity of less than 80%, cancer, patients without PFT values at stable state and patients improving with only NIV.

All the patients were ventilated using conventional respirators in the assist control (A/C) mode. The weaning procedure was standardized. Weaning trials were performed switching the ventilator to the pressure support ventilation mode, with a peak inspiratory pressure (PIP) between 12 to 20 cm H_2_O to maintain an expiratory tidal volume (Vt) about 5 ml.kg ^-1^, a respiratory rate between 20 to 30/min and an arterial oxygen saturation (SaO_2_) > 93%. As soon as PIP reached 12 cm H_2_O with a good tolerance, a test of spontaneous ventilation with a T-tube was performed. In case of failure, the test was repeated every day. If the patient was able to breathe for 120 minutes without mechanical support, extubation was performed.

Patients were divided into two separate groups for data analysis, those weaned from MV (Group A), and those not weaned (Group B). Weaning success was defined as breathing autonomy during at least 48 h after extubation with SpO_2_ > 90and the absence of signs of muscular weakness [7]. Weaning failure was defined as death while on MV or failure to be disconnected from the respirator despite repeated weaning trials during at least 30 days [8].

### Mechanical ventilation

NIV was used before intubation in 20 patients (80%). Immediate EI for MV was performed in 5 patients (20%) because of comatose (n = 4) or near fatal acute respiratory failure with tachypnea and paradoxal breathing (n = 1). When used, initial NIV was performed in pulmonary medicine unit (n = 2, 10%), emergency unit (n = 12, 60%) or ICU (n = 6, 30%). NIV was performed using conventional respirators: Legendair® (Airox), Evita® (Dräger) or Engström® (General Electric) in the PIP + positive expiratory pressure (PEP) mode with a naso-buccal mask. The failure of the non invasive approach was related to exhaustion (n = 15, 75%), occurrence or persistence of coma (n = 5, 25%), or intolerance of NIV with major anxiety, agitation, or vomitis (n = 3, 15%). In invasive approach, all patients were ventilated with Evita® or Engström® in the A/C mode. The median length of invasive MV was 9 days (2–90) in Group A, and 20 days (8–42) in Group B (p = 0.04). In group A, a tracheotomy was performed in 3 patients.

### Data collection

At inclusion, demographic data (age, sex, weight, height, body mass index), nutritional risk index (NRI), albumin, prealbumin, arterial blood gases, echocardiographic results for left ventricular ejection fraction (LVEF) and systolic pulmonary arterial pressure (PAPs) and the simplified acute physiology score (SAPS_II_) were systematically recorded. Daily activity was assessed at admission using the activities of daily living score (ADL) [7]. This score is based on 6 criteria: 1) bathing with sponge, bath, or shower, 2) dressing, 3) toilet use, 4) transferring (in and out of bed or chair), 5) urine and bowel continence, 6) eating. The index ranks adequacy of performance in these six functions with a yes/no score for each of the six functions. A score of 6 indicates full function, 4 indicates moderate impairment, and 2 or less indicates severe functional impairment.

Initial use of NIV, cause of acute respiratory failure (acute bronchitis, pneumonia, pulmonary embolism…), Logistic Organ Dysfunction System (LODS) score and all medical events occurring during hospitalization (toxic shock, nosocomial infections, respect of nutritional target, curare using) were recorded. Weaning success or failure and its term were noted.

The PFT and arterial blood gases obtained from a phase of clinical stability before ARF were also recorded.

The study was approved by the Institutional Review Board at the University Hospital of Reims (France), and patient consent was waived.

Two authors (K Langlet and G Deslee) had full access to all the data in the study and take responsibility for the integrity of the data and the accuracy of the data analysis, including and especially any adverse effects.

### Data analysis

The Kaplan-Meier method was used to analyse survival. Statistical analysis to compare the difference between the “weaned” and “not weaned” group was performed using Fischer Test, Kruskal-Wallis test, Spearman and Tau Kendall test. This statistical analysis was retrospective.

A p-value of less 0.05 was considered significant.

## Results

### Patients

**Figure 1:**
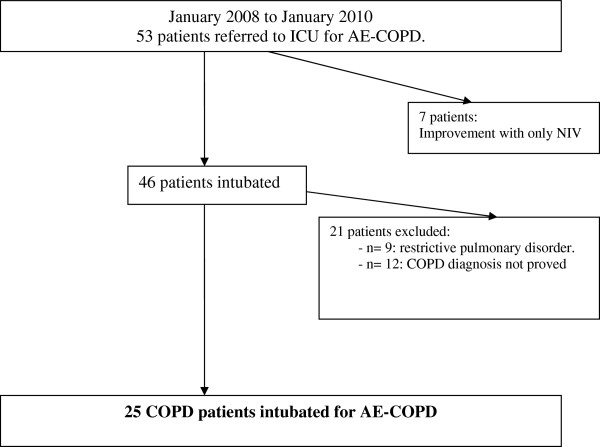
Studied and excluded patients.

Between January 2008 to January 2010, 53 patients were admitted in the intensive care unit (ICU) of the Saint Philibert Hospital in France for ARF. Seven patients (13.2%) improved with NIV and were not intubated. Among the 46 patients requiring invasive MV, 9 (19.5%) were excluded because of a restrictive pulmonary disorder related to obesity (n = 7, 78%) or tuberculosis sequelae (n = 2, 22%). Twelve patients (26%) were excluded because of absence of PFT performed before admission in ICU, then the diagnosis of COPD before respiratory failure was not possible to establish. Therefore, 25 patients were considered in the data analysis (Figure 1). The characteristics of the patients included are presented in Table 1. Failure to wean occurs in 32% of patients (n = 8). The median age was 68 years (49–81), there were 24% of females and 76% of males. Median SAPS_II_ was 39 (22–79). Median ADL score was 3.7. The severity of COPD according to GOLD was a follows: 46% GOLD IV (very severe), 16% GOLD III (severe), 38% GOLD II (moderate). Median ratio PaO_2_/FiO_2_ was 220 (22–250). Median pH at admission was 7.25 (7.07-7.39), median PCO_2_ was 75 mmHg (50–114). The mains causes of respiratory failure were acute bronchitis (n = 12, 48%), pneumonia (n = 9, 36%) and pulmonary embolism (n = 2, 8%). Median BMI was 25 kg /m^2^ (18–37). Median NRI was 82.9 (66–102). According to these results, 92% of the patients were undernourished and 56% had malnutrition.

**Table 1:** Comparison of simple parameters recorded before endo-tracheal intubation in weaned and not weaned groups

	**Total (n = 25)**	**Group A (n = 17)**^**a**^	**Group B (n = 8)**^**a**^	**P**
		**Weaned**	**not weaned**	
***General characteristics***				
Age,yr ^b^	68 [49–81]	68 [49–81]	68.5 [51–77]	0.52
M/F	19/6	13/4	6/2	0.68
SAPS _II_^b^	39 [22–79]	39 [28–79]	38.5 [22–69]	0.69
***Pulmonary status during a phase of clinical stability***				
FEV_1_ l ^b^	0.66 [0.220-2.50]	0.710 [0.220-2.50]	0.630 [0.32-1.47]	0.60
FEV_1_predicted ^b^	30 [11–80]	31 [11–80]	26.5 [15–50]	0.33
PaO_2_ mmHg ^b^	69 [41–95]	69 [50–95]	69 [41–86]	0.52
PaCO_2_ mmHg ^b^	49 [30–91]	48 [30–91]	54 [40–61]	0.37
pH ^b^	7.40 [7.28-7.50]	7.40 [7.33-7.50]	7.38 [7.28-7.45]	0.84
***Arterial blood gases at admission***				
PaO_2_/FiO_2_^b^	220 [22–250]	220 [22–250]	157 [120–250]	0,57
PaCO_2_ mmHg ^b^	75 [50–114]	74.5 [56–114]	76 [50–102]	0,46
pH ^b^	7.25 [7.07-7.39]	7,29 [7.07-7.37]	7,24 [7.08-7.39]	0.49
***Cardiac echography at admission***				
LVEF ^b^	60 [30–70]	60 [40–70]	50 [30–65]	0.26
PAPs ^b^	45 [25–80]	45 [25–65]	50 [30–80]	0.45
***Cause of respiratory failure***				
Acute exacerbation ^a^	12 (48%)	8 (47%)	4 (50%)	0.63
Pneumonia ^a^	9 (36%)	5 (30%)	4 (50%)	0.37
Pulmonary embolism ^a^	2 (8%)	2 (11.5%)	0 (0%)	0.43
Other ^a^	2 (8%)	2 (11.5%)	0 (0%)	0.43
***Nutritional status***				
BMI ^b^	25 [18–37]	25 [19–36.7]	25 [18–37]	0.86
NRI ^b^	82.9 [66–102]	82.8 [66–93]	80.4 [73–102]	0.73
Albumin ^b^	27 [20–38]	27 [20–34]	27.5 [21–38]	0.49
Prealbumin ^b^	195 [74–400]	215 [130–400]	164 [74–337]	0.50
***Corticoïds***^a^	3 (12%)	2 (11.8%)	1 (12.5%)	0.72
***ADL score***^*c*^	4.6 ± 1.1	5,1 ±1.1	3.7 ± 0.7	0.006

^a^ data are expressed as numbers (%); ^b^ data are expressed as median [minimum – maximum] ^c^: mean ± SD.

*M* male, *F* female.

### Prognosis factors

**Figure 2:**
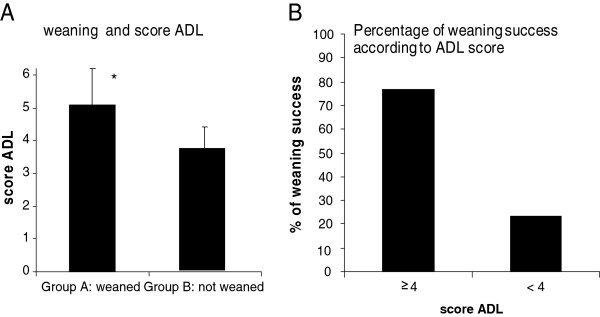
**A. Weaning and ADL score. B.** Percentage of weaning success according to ADL score.

Next, we investigated the relationships between demographic characteristics, PFT, arterial blood gas, causes of respiratory failure, nutritional status, corticosteroids treatments, cardiac assessment and the probability for the patient to be weaned (Group A) or not (Group B) from MV (Table 1). In this study, the only variable which was correlated with weaning success was the ADL score (respectively 5.1 ±1.1 versus 3.7 ± 0.7 p < 0.01) (Figure 2A). Interestingly, weaning was achieved in 76.5% of the cases with an ADL score ≥ 4 and in 23.5% of the cases with an ADL score < 4 (p < 0.05) (Figure 2B).

Next, we investigated the relationships between the occurrence of nosocomial infections, failure of organs, curare use and the probability for the patient to be weaned. 11.8% of patients in group A were treated by curare, 12.5% in group B, the difference was no significant. The rate of nosocomial infection was significantly higher in the group B (p < 0.01), whereas organs failure and the use of curare did not reach statistical significance. Nosocomial infections were pneumonia (n = 5, 50%), urinary infections (n = 3, 30%) and bacteraemia (n = 2, 20%). The most frequently identified pathogens were *Pseudomonas aeruginosa* (n = 4, 40%), *Escherichia coli* (n = 3, 30%), *Staphylococcus aureus* and *Staphylococcus epidermidis* (n = 2, 20%).

### Long-term outcome

**Figure 3:**
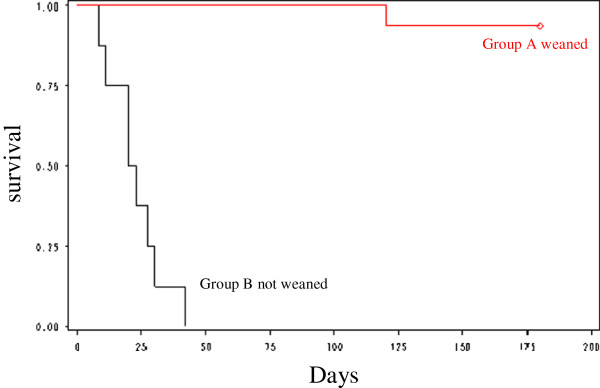
Kaplan-Meier Survival curve of the weaned and not weaned groups.

Informations regarding survival and the ADL score were collected by phone six months later. Survival data in the weaned group and not weaned group are shown in Figure 3. At six months, the overall rate of mortality was 36%. All the patients of group B died within 42 days after ICU admission with a median delay of 22 days (8–42). No patient died after weaning during the hospitalization in ICU. One patient was readmitted in ICU within 6 months for AE-COPD and died during this hospitalization. 41% of the surviving patients were readmitted for AE-COPD in pulmonary medicine unit within 6 months. 12% of the patients have been hospitalized in rehabilitation centre before being discharged at home. 18% were treated by nocturnal non invasive ventilation at home at 6 months. The median ADL score of surviving patient was performed at six months (5.1 versus 4.3, p <0.05).

The predictive factors of mortality at 6 months were the ADL score at admission (ADL ≤ 4, p = 0.014) and nosocomial infection (p = 0.008).

## Discussion

This prospective observational long-term monocenter study analysed the predictive factors of ventilator weaning and mortality in a series of 25 COPD patients with ARF requiring EI for mechanical ventilation. Our results demonstrated that a low ADL score (≤4) at admission correlates with the risk of weaning failure and mortality at 6 months. Our study did not identify any other simple clinical, functional or biological parameters at admission associated with the risk of weaning failure or mortality.

In our study, the diagnosis and the severity of COPD were very carefully investigated and were based on PFT results obtained at stable state before admission in ICU, allowing to exclude other causes of chronic respiratory diseases than COPD. Moreover, the same type of treatment, mechanical ventilation and respirator weaning procedure were applied to all patients in this monocenter study. Most of the patients (80%) had been treated by NIV before intubation as currently recommended [9]. The intubation for MV was based on definite clinical and blood gas criteria which were correlated with a high rate of NIV failure (respiratory frequency >35/mn, pH < 7.25 at admission [10], coma [11]). None of the patients was weaned within the first 48 h, suggesting that the indication of intubation was appropriate. The nutritional status was very precarious with 92undernourished and 56with malnutrition. The degree of denutrition is more important in our series than in other studies published before the use of NIV [2-4], which could be related to the selection of more severe patients not responding to NIV in our study.

The prognosis of patients with ARF requiring EI for mechanical ventilation is poor. In this study, the mortality rate at 6 months is of 36%, which is in accordance with previous series [2-4]. Of notes, almost all the patients (8/9) are died not weaned. Several explanations can be proposed to account for difficulties of weaning and mortality: terminal stage of respiratory insufficiency, impaired pulmonary function, muscular weakness, hypercapnia [12-14]. However, the prediction of outcome after invasive mechanical ventilation of COPD patients remains a difficult challenge. The value of FEV_1_ as predictor of weaning difficulties has been investigated in different studies with different results. Menzies and al [3] showed a significant correlation between weaning difficulties and mortality at one year and FEV_1_ impairment, whereas other studies did not find any correlation with FEV_1_[15]. In our study, we did not find any correlation between FEV_1_ or any clinical or functional data at inclusion and the outcome COPD patients treated by invasive MV. Menzies and al [3] showed that a model involving eight parameters (fever, FEV_1_/FVC, age, premorbid hypercapnia, hyperleukocytosis, number of previous intubation, low-flow oxygen treatment, plasma protein level) was able to predict outcome in 78of AE-COPD treated by invasive mechanical ventilation.

We found an association between weaning failure and nosocomial infection. However, it must be pointed out that this does not imply a causal relationship. It can be assumed that patients who fail to wean have an increased duration of MV and require higher levels of sedation. Moreover, the risk for nosocomial infection may be a consequence of poor status at admission. In this study, the occurrence of nosocomial infection associated with failure organs led to stop active therapy. The do-not-resuscitate decision was warranted by the reject or the disability to perform a tracheotomy. Almost all patients (8/9), who failed to wean died and no patient has been stabilized by chronic intermittent ventilation.

The use of curare might be responsible of more frequent failure of weaning, even if it is not statistically significant in our study involving a limited number of patients. The use of curare is more frequent when the situation is more severe, leading to a potential bias as for its association with the failure of weaning. Nevertheless, toxicity of curare on neuromuscular function is known, slowing the weaning, especially when they are used for a long time.

In our study, the only parameter identified at admission as predictive factor of both weaning success and long-term survival was the premorbid level of activity assessed by the ADL score. An ADL score ≥ 4 was associated with 76.5of weaning success, whereas an ADL < 4 was associated with only 23.5of weaning success. Interestingly, a low ADL score (ADL < 4) was also a strong predictor of mortality at 6 months. As suggested by other studies, the premorbid level of activity is influenced by many factors involving clinical and functional parameters but also unmeasurable factors such as motivation, family support or other psychosocial factors [3,16]. It must be pointed out that the ADL score investigated in this study is a very simple and easy-to-use score in clinical practise. However, our study has several limitations which do not permit to come to a definitive conclusion regarding the interest of the ADL score in clinical practise. The number of patients included in this monocenter study is small and our results have to be confirmed in additional larger multicenter studies. Moreover, the rate of 23.5of weaning success in patients with a low ADL score (<4) reported in this study is not ethically acceptable to be used as a single parameter in the decision to use or not EI in ARF of COPD patients requiring invasive MV.

## Conclusion

Our prospective monocenter study involving 25 consecutive ARF in COPD patients requiring invasive MV confirmed the difficulty to determine predictive factors of success or failure of weaning and mortality. At admission, only the ADL score was predictive of weaning success and mortality at 6 months. Our results suggest that the assessment of daily activities should be an important component of ARF management in COPD patients. This work is a pilot study and further studies are needed to investigate the ADL score and other potentially more accurate scores of daily activities in clinical practice.

## Abbreviations

A/C mode: Assist Control mode; ADL score: Activities Daily Living score; AECOPD: Acute Exacerbations of Chronic Obstructive Pulmonary Disease; BMI: Body Mass Index; COPD: Chronic Obstructive Pulmonary Disease; EI: Endotracheal intubation; FEV_1_: Forced Expiratory Volume in one second; FVC: Forced Vital Capacity; GOLD: Global Initiative for chronic Obstructive Lung Disease; ICU: Intensive Care Unit; LODS: Logistic Organ Dysfunction System; LVEF: Left Ventricular Ejection fraction; MV: Mechanical ventilation; NIV: Non Invasive Ventilation; NRI: Nutritional Risk Index; PAPs: Systolic Pulmonary Arterial Pressure; PEP: Positive Expiratory Pressure; PFT: Pulmonary Function Test; PIP: Peak Inspiratory Pressur; SAPS_II_: Simplified Acute Physiology Score 2; Vtm: tidal Volume.

## Competing interest

None of the authors of the present manuscript have a commercial or other association that might pose a competing interest.

## Authors' contributions

KL, VT, FC, CP, KN, BC, Fl and GD conceived the study. KL acquired data. CB performed the statical analysis. KL and GD drafted the manuscript. All authors read and approved the manuscript prior to submission.

## Pre-publication history

The pre-publication history for this paper can be accessed here:

http://www.biomedcentral.com/1471-2466/12/66/prepub

## References

[B1] Ram FS Picot J Lightowler J Wedzicha JA Non invasive positive pressure ventilation for treatment of respiratory failure due to exacerbations of chronic obstructive pulmonary disease Cochrane Database Syst Rev 2004 CD 004104 10.1002/14651858.CD004104.pub3 15266518

[B2] Nava S Rubini F Zanotti E Ambrosino N Bruschi C Vitacca M Fracchia C Rampulla C Survival and prediction of successful ventilator weaning in COPD patients requiring mechanical ventilation for more than 21 days Eur Respir J 1994 7 9 1645 1652 10.1183/09031936.94.07091645 7995395

[B3] Menzies R Gibbons W Goldberg P Determinants of weaning and survival among patients with COPD who require mechanical ventilation for acute respiratory failure Chest 1989 95 2 398 405 10.1378/chest.95.2.398 2914493

[B4] Kaelin RM Assimacopoulos A Chevrolet JC Failure to predict six-month survival of patients with COPD requiring mechanical ventilation by analysis of simple indices. A prospective study Chest 1987 92 6 971 978 10.1378/chest.92.6.971 3677842

[B5] Carlucci A Delmastro M Rubini F Fracchia C Nava S Changes in the practice of non–invasive ventilation in treating COPD patients over 8 years Intensive Care Med 2003 29 3 419 425 12624663 10.1007/s00134-002-1574-1

[B6] Pauwels RA Buist AS Calverley PMA Jenkins CR Hurd SS Global strategy for the diagnosis, management and prevention of COPD: NHLBI/WHO Global Initiative for Chronic Obstructive Lung Disease (GOLD) Workshop Summary Am J Resp Crit Care Med 2001 163 5 1256 1276 Updated 2007 11316667 10.1164/ajrccm.163.5.2101039

[B7] Katz S Ford AB Moskowitz RW Jackson BA Jaffe MW Studies of illness in the aged. The index of ADL: a standardized measure of biological and psychosocial function JAMA 1963 185 914 919 10.1001/jama.1963.03060120024016 14044222

[B8] Société de réanimation de langue française Sevrage de la ventilation mécanique (à l’exclusion du nouveau-né et du réveil d’anesthésie). XXIème conférence de consensus de la SRLF Reanimation 2001 10 697 698

[B9] Elliott MW Non-invasive ventilation in acute exacerbations of chronic obstructive pulmonary disease: a new gold standard? Intensive Care Med 2002 28 12 1691 1694 10.1007/s00134-002-1503-3 12580151

[B10] Anton A Güell R Gomez J Serrano J Castellano A Carrasco JL Sanchis J Predicting the result of non-invasive ventilation in severe acute exacerbations of patients with chronic flow limitation Chest 2000 117 3 828 833 10.1378/chest.117.3.828 10713013

[B11] Wyatt J Bellis F British Thoracic Society guidelines on non-invasive ventilation Thorax 2002 57 3 192 211 12205000 10.1136/emj.19.5.435 PMC1725985

[B12] Rochester DF Martin LL Respiratory muscle rest The Thorax N York, Dekker 1985 43 1303 1328

[B13] Roussos C Respiratory muscle fatigue in the hypercapnic patient Bull Eur Physiopathol Resp 1979 15 117 126 554695

[B14] Laghi F D’alfonso N Tobia MJ Pattern of recovery from diaphragmatic fatigue on 24 h J Appl Physiol 1995 79 2 539 546 7592215 10.1152/jappl.1995.79.2.539

[B15] Creutzberg E Fokkens B Wouters E Staging COPD by airflow obstruction alone underestimates impaired health status Am J Respir Crit Care Med 2002 165 A654

[B16] Jessen O Kristensen HS Rasmussen K Tracheostomy and artificial ventilation in chronic lung disease Lancet 1967 2 7505 9 12 4165457 10.1016/s0140-6736(67)90056-6

